# Correction: Molecular basis of ligand-dependent Nurr1-RXRα activation

**DOI:** 10.7554/eLife.107190

**Published:** 2025-04-14

**Authors:** Xiaoyu Yu, Jinsai Shang, Douglas J Kojetin

**Keywords:** None

Yu X, Shang J, Kojetin DJ. 2023. Molecular basis of ligand-dependent Nurr1-RXRα activation. *eLife* **12**:e85039. doi: 10.7554/eLife.85039.

Published 27 April 2023

After publication, we discovered that the RXR antagonist ligand PA452 was incorrectly spelled as ‘PA425’ throughout most of the manuscript.

After publication, we discovered that the RXR antagonist ligand PA452 was incorrectly spelled as ‘PA425’ throughout most of the manuscript.

We also noticed that the statistical testing reported in the legend for Figure 4b (RXRα LBD TR-FRET coactivator peptide interaction assay) was incorrectly applied (Brown-Forsythe and Welch multiple comparisons) because a Shapiro-Wilk normality test in GraphPad Prism (v.10.4.1) shows that the data can be compared assuming equal standard deviations (ordinary one-way ANOVA tests for multiple comparisons with Dunnett corrections).

Finally, we discovered two errors in Figure 4b and Figure 4—source data 1 that resulted from copy-and-paste of the wrong RXRα LBD TR-FRET values for two ligand treated conditions. Data reported for LG100754 correspond to the data values LG100268, an accidental copy-and-paste error. Data reported for LG100268 were accidentally duplicated from the values for another ligand (bexarotene), another accidental copy-and-paste error. These copy-and-paste errors propagated into the correlation analyses in Figure 4e, Figure 4g, and Figure 7, but only have a small impact on the Spearman and Pearson correlation coefficients and PCA analysis.None of these corrections change the conclusions of the paper. We sincerely apologize for these errors. The specific errors and needed corrections are described below.

We corrected all incorrectly spelt instances of PA452 throughout the article including in the main text, Figures, Tables, and Source data.

Corrected Figure 4b legend text:

Statistical testing was performed and p-values were calculated using ordinary one-way ANOVA tests for multiple comparisons with Dunnett corrections relative to DMSO control treated condition.

Original Figure 4b legend text:

Statistical testing was performed and p-values were calculated using the Brown-Forsythe and Welch multiple comparisons test relative to the DMSO control treated condition.

Corrected Figure 4—source data 1, Excel file rows for: LG100754:

mean, 0.583; s.d., 0.02778489; replicates, [0.569, 0.565, 0.615]

LG100268: mean, 2.788; s.d., 0.04680812; replicates, [2.842, 2.759, 2.763]

Original Figure 4—source data 1, Excel file rows for:

LG100754: mean, 2.788; s.d., 0.04680812; replicates, [2.842, 2.759, 2.763]

LG100268: mean, 2.865666667; s.d., 0.010263203; replicates, [2.877, 2.863, 2.857]

The corrected Figure 4 is shown here:

**Figure 1:**
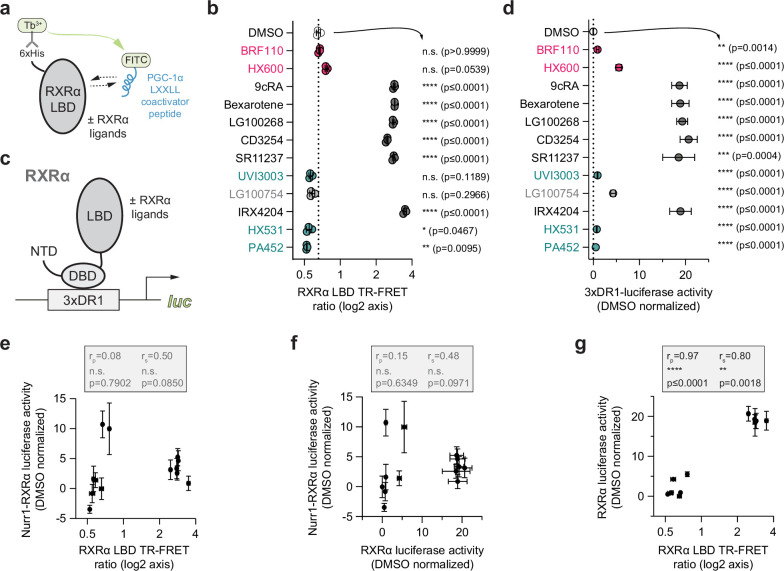


The originally published Figure 4 is shown for reference:

**Figure 2:**
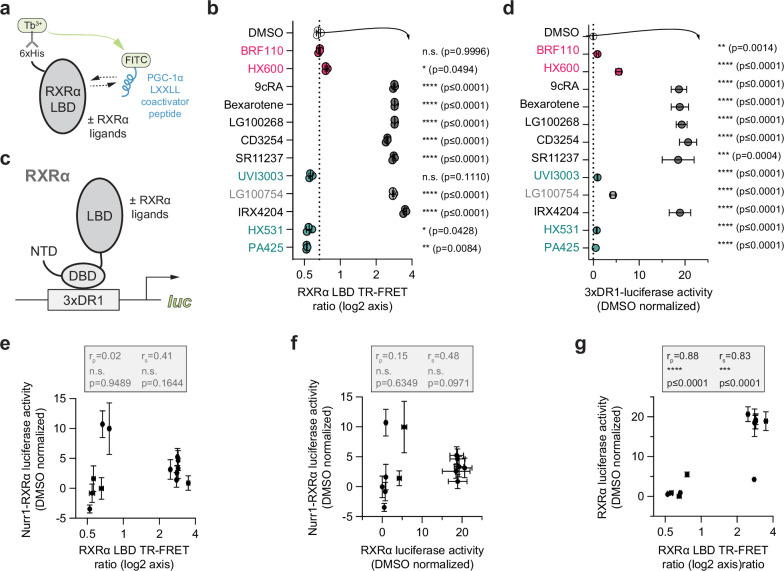


The corrected Figure 7 is shown here:

**Figure 3:**
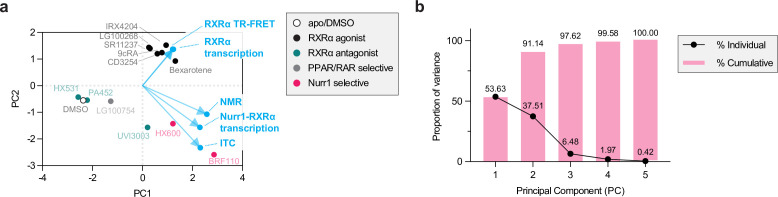


The originally published Figure 7 is shown for reference:

**Figure 4:**
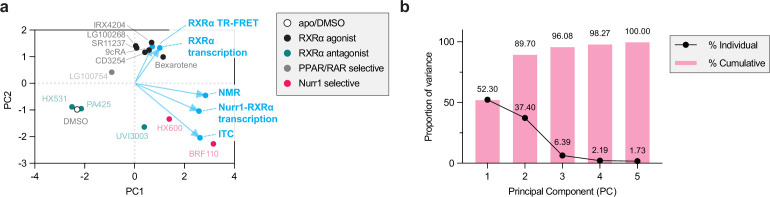


The article has been corrected accordingly.

