# Supplementary figures and images for: Molecular basis of ligand-dependent Nurr1-RXRα activation
Source: eLife. 2023 Apr 27;12:e85039. doi: 10.7554/eLife.85039 (PMC10259986; doi:10.7554/eLife.85039)

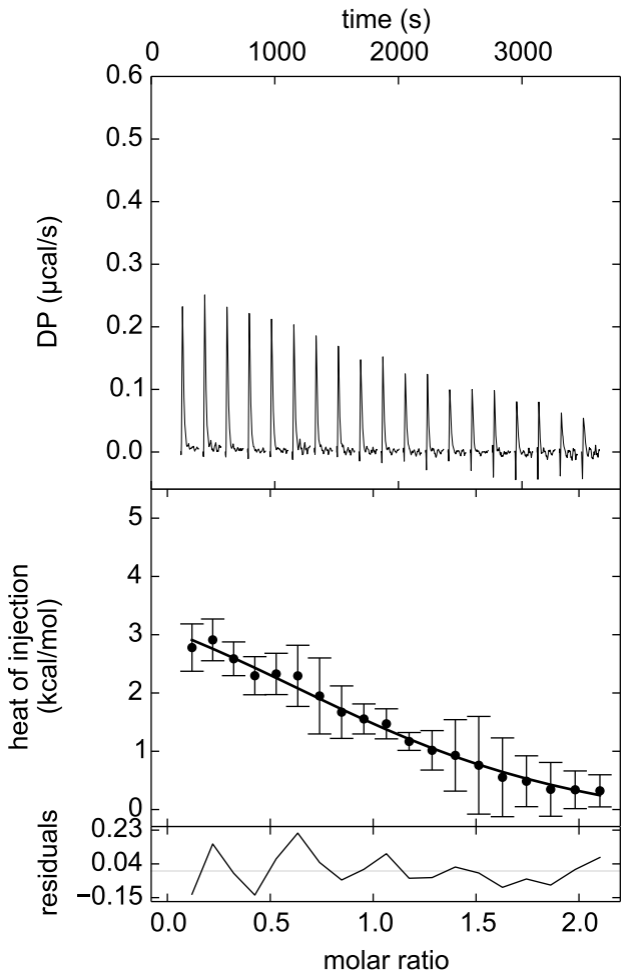

Supplement: Figure 5—source data 1. [file elife-85039-fig5-data1.zip › Figure 5-source data 1/BRF110.pdf]

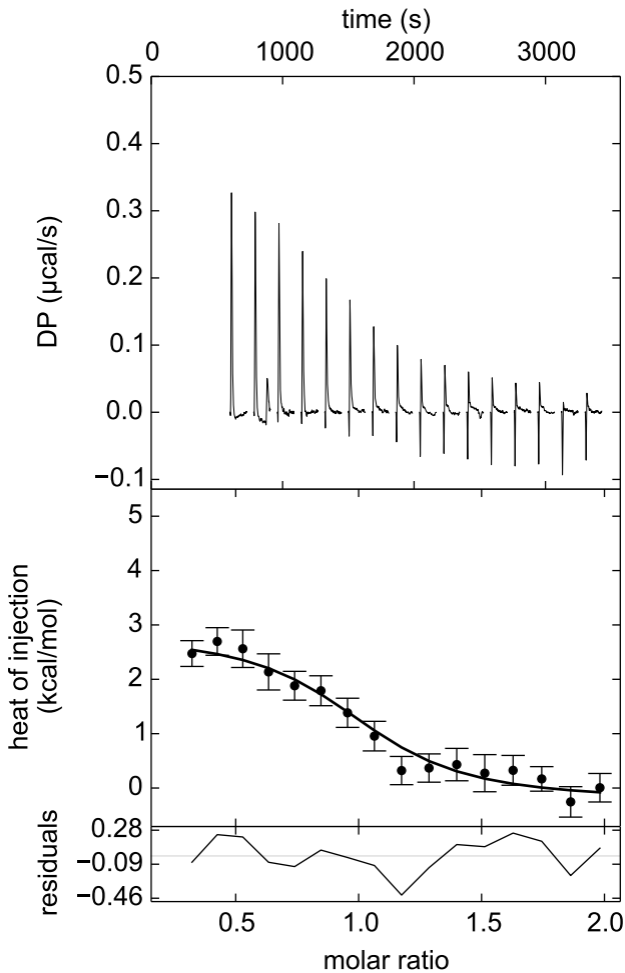

Supplement: Figure 5—source data 1. [file elife-85039-fig5-data1.zip › Figure 5-source data 1/HX531.pdf]

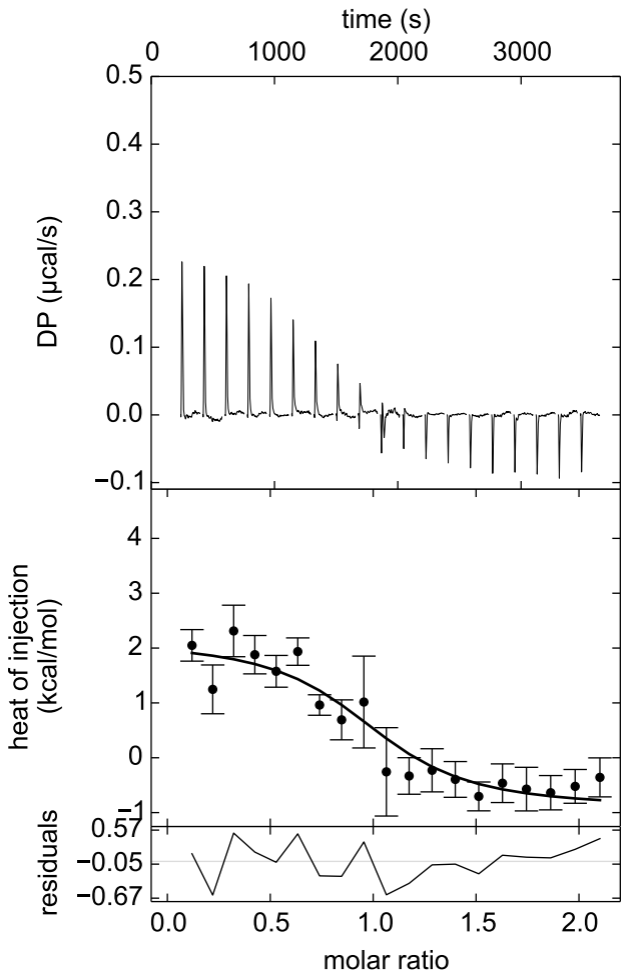

Supplement: Figure 5—source data 1. [file elife-85039-fig5-data1.zip › Figure 5-source data 1/Danthron.pdf]

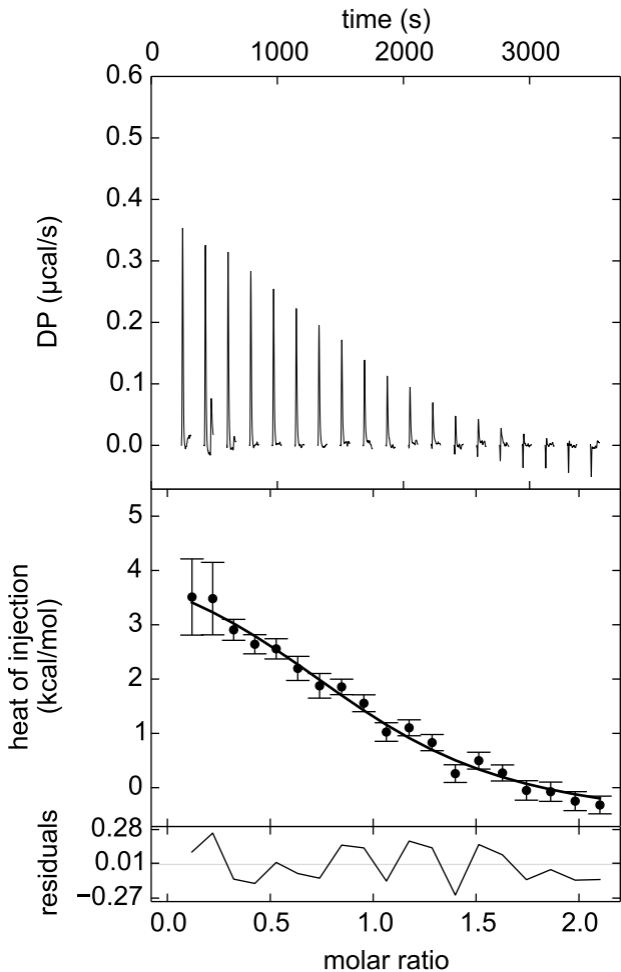

Supplement: Figure 5—source data 1. [file elife-85039-fig5-data1.zip › Figure 5-source data 1/9cRA.pdf]

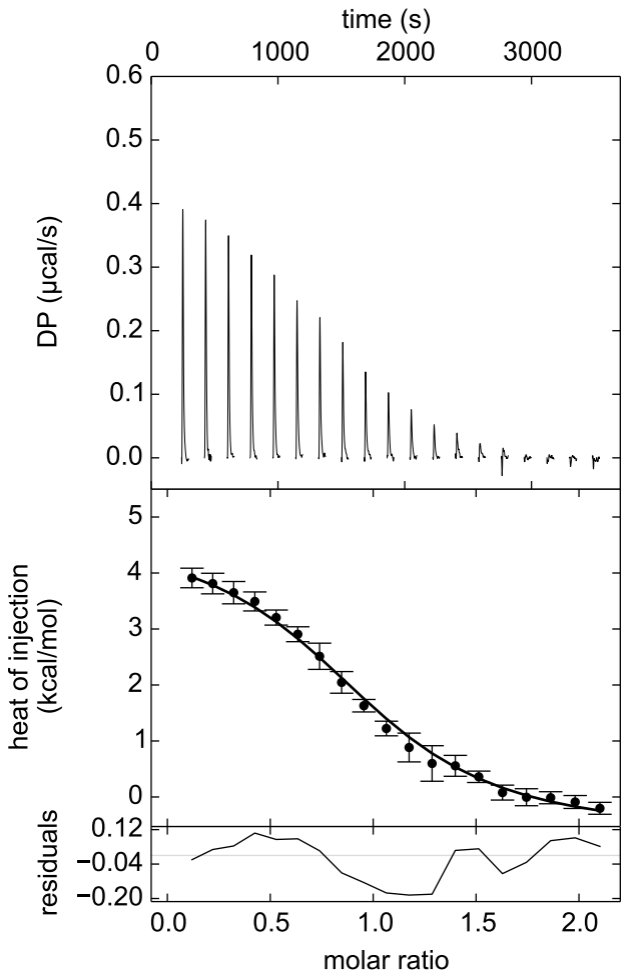

Supplement: Figure 5—source data 1. [file elife-85039-fig5-data1.zip › Figure 5-source data 1/PA452.pdf]

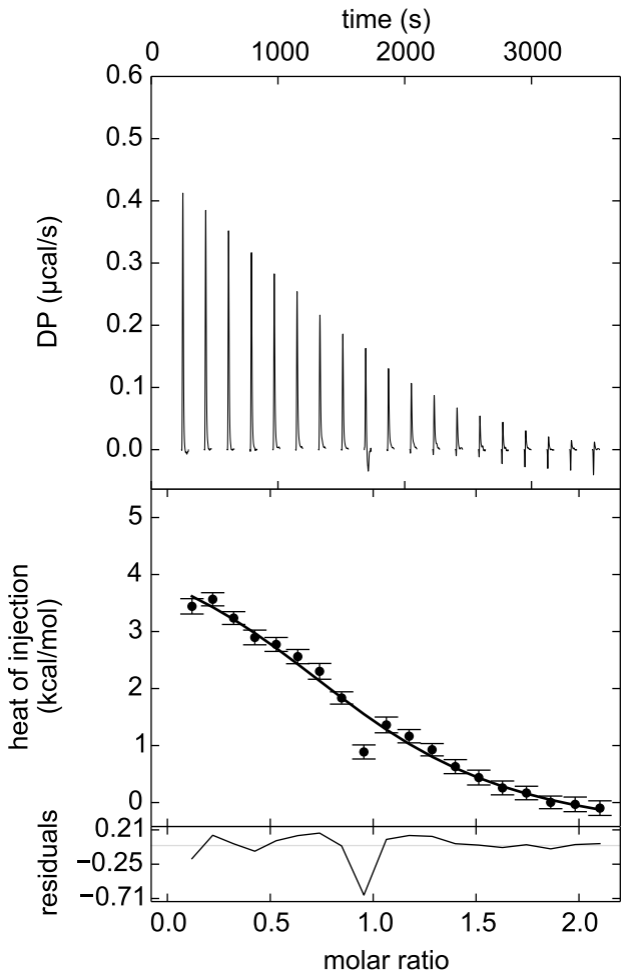

Supplement: Figure 5—source data 1. [file elife-85039-fig5-data1.zip › Figure 5-source data 1/CD3254.pdf]

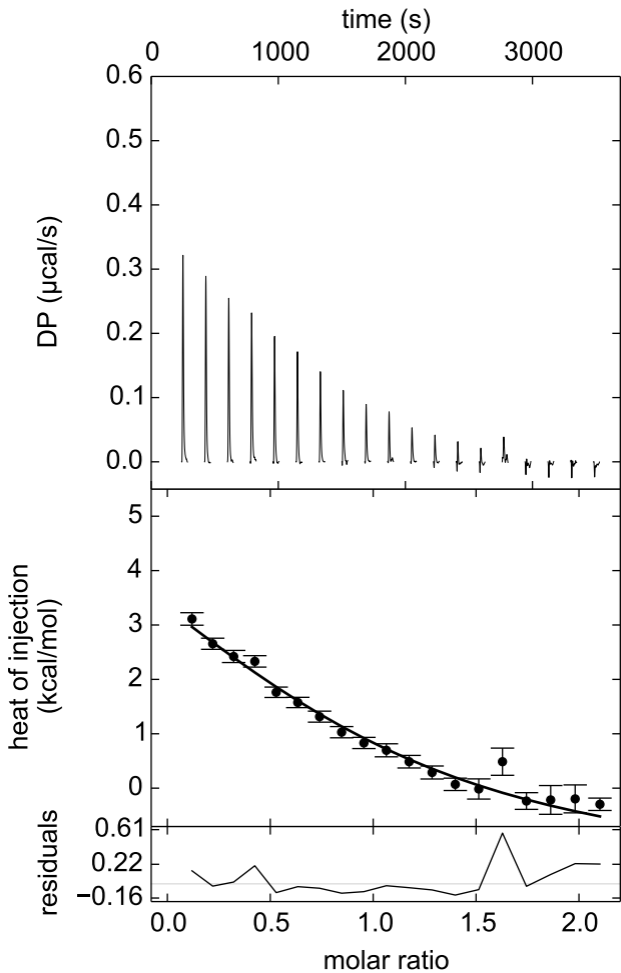

Supplement: Figure 5—source data 1. [file elife-85039-fig5-data1.zip › Figure 5-source data 1/Bexarotene.pdf]

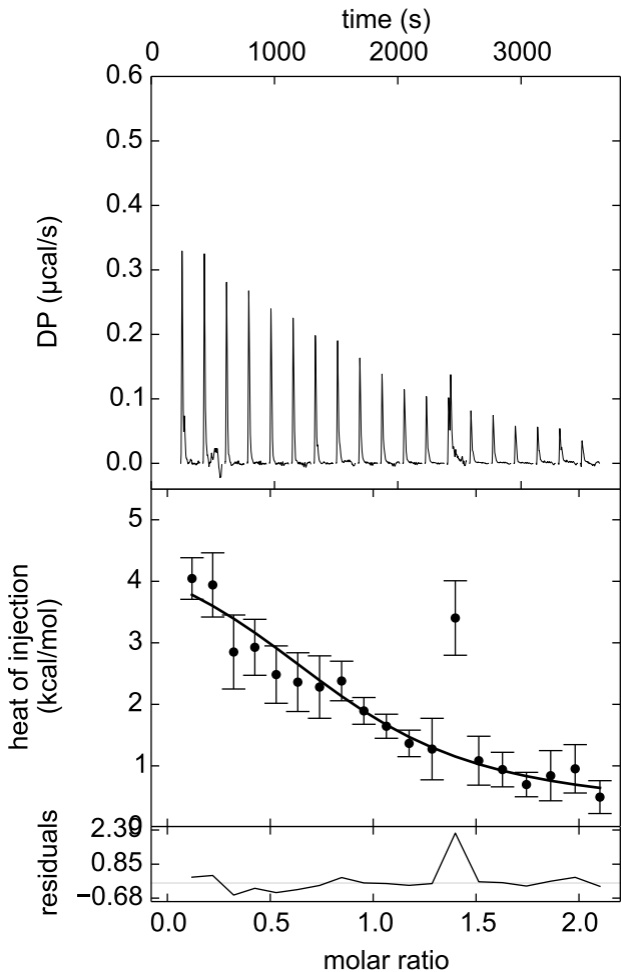

Supplement: Figure 5—source data 1. [file elife-85039-fig5-data1.zip › Figure 5-source data 1/HX600.pdf]

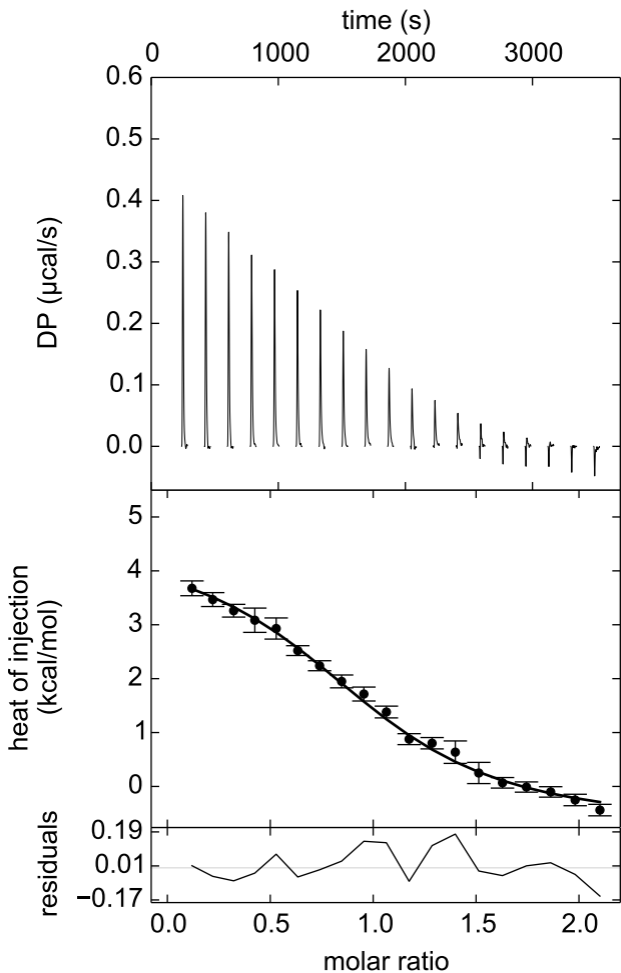

Supplement: Figure 5—source data 1. [file elife-85039-fig5-data1.zip › Figure 5-source data 1/SR11237.pdf]

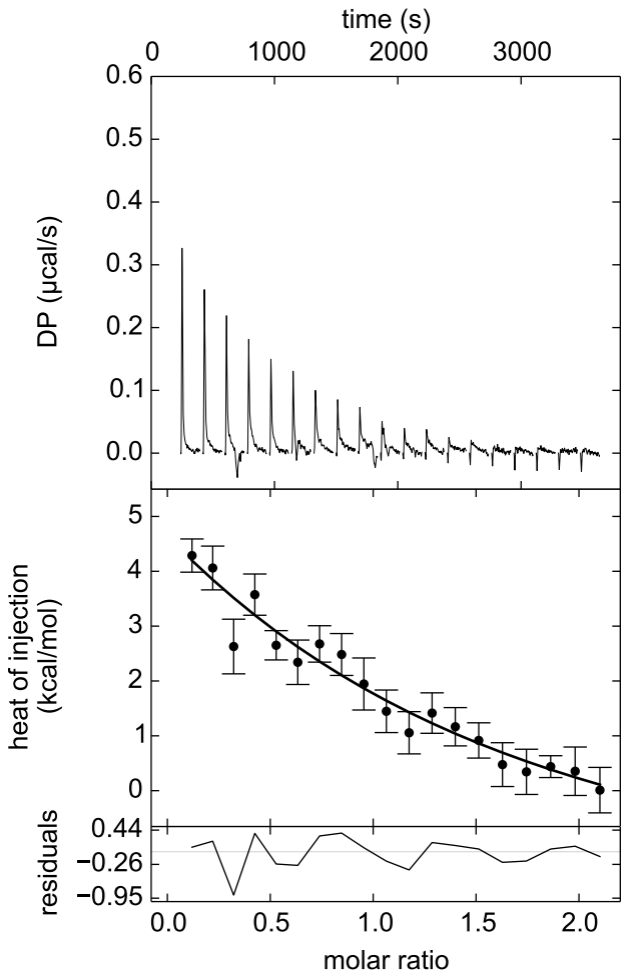

Supplement: Figure 5—source data 1. [file elife-85039-fig5-data1.zip › Figure 5-source data 1/UVI3003.pdf]

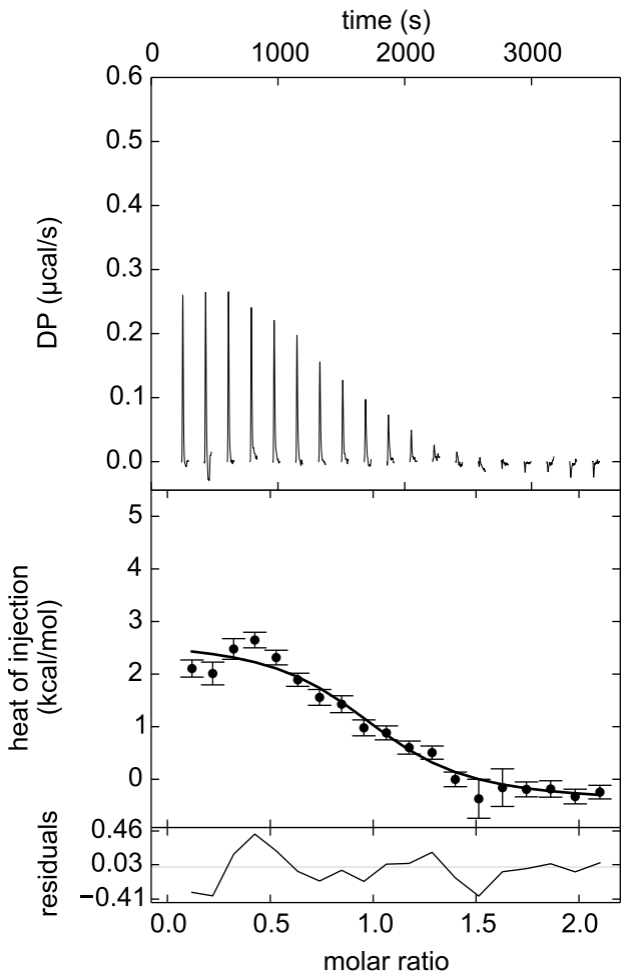

Supplement: Figure 5—source data 1. [file elife-85039-fig5-data1.zip › Figure 5-source data 1/Rhein.pdf]

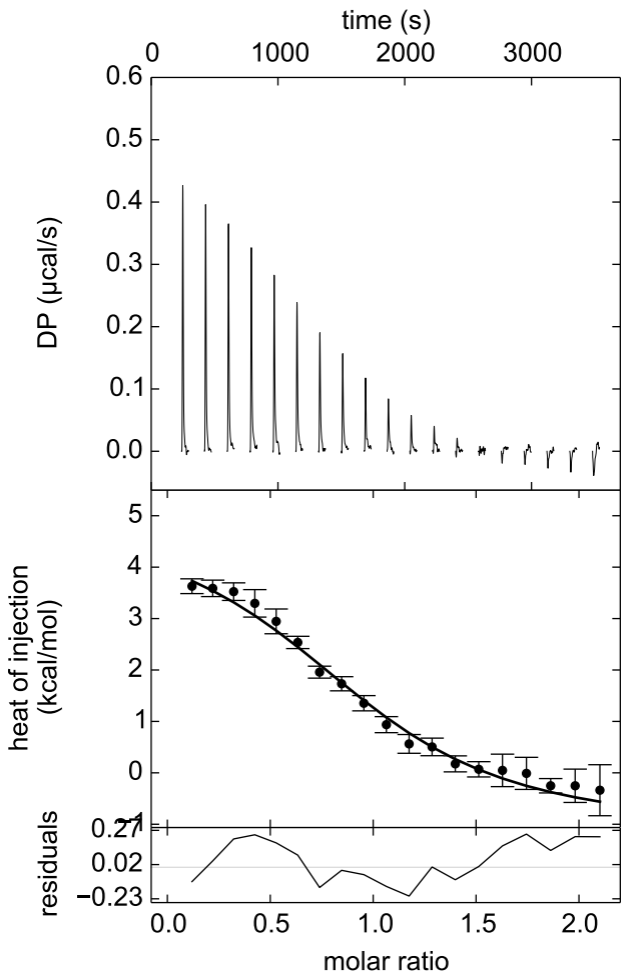

Supplement: Figure 5—source data 1. [file elife-85039-fig5-data1.zip › Figure 5-source data 1/LG100754.pdf]

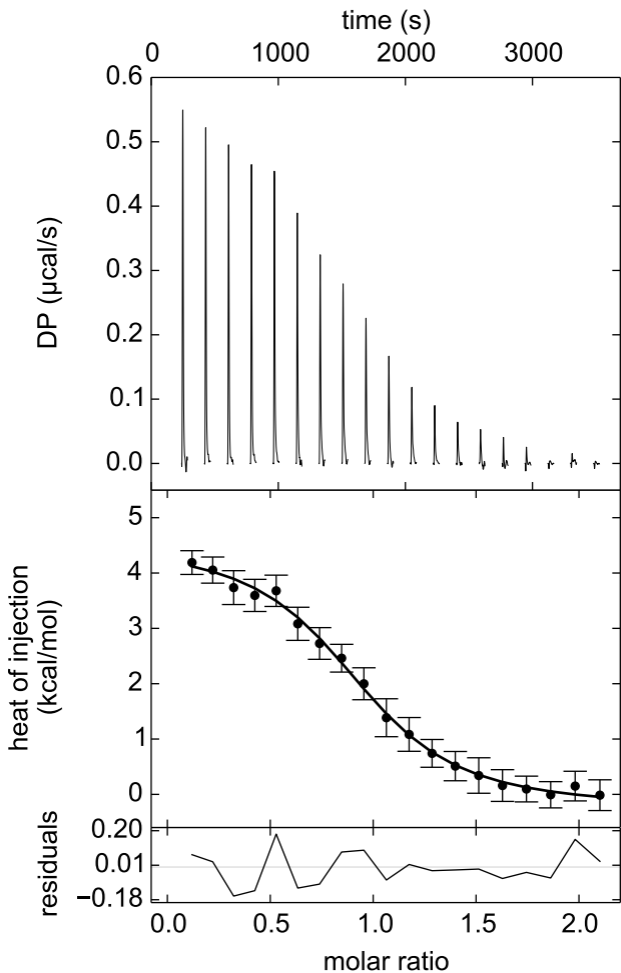

Supplement: Figure 5—source data 1. [file elife-85039-fig5-data1.zip › Figure 5-source data 1/DMSO.pdf]

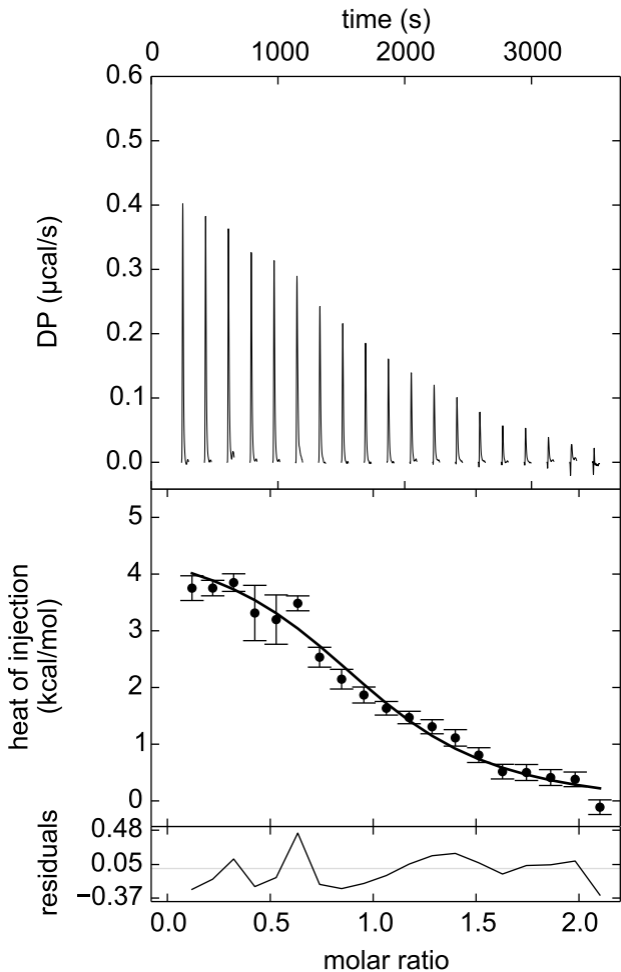

Supplement: Figure 5—source data 1. [file elife-85039-fig5-data1.zip › Figure 5-source data 1/LG100268.pdf]

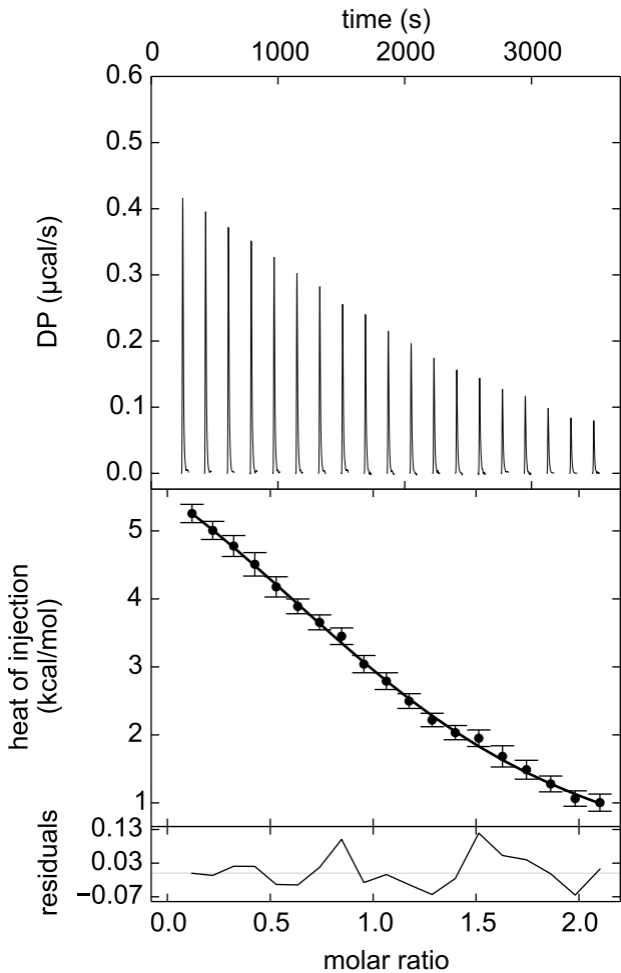

Supplement: Figure 5—source data 1. [file elife-85039-fig5-data1.zip › Figure 5-source data 1/IRX4204.pdf]

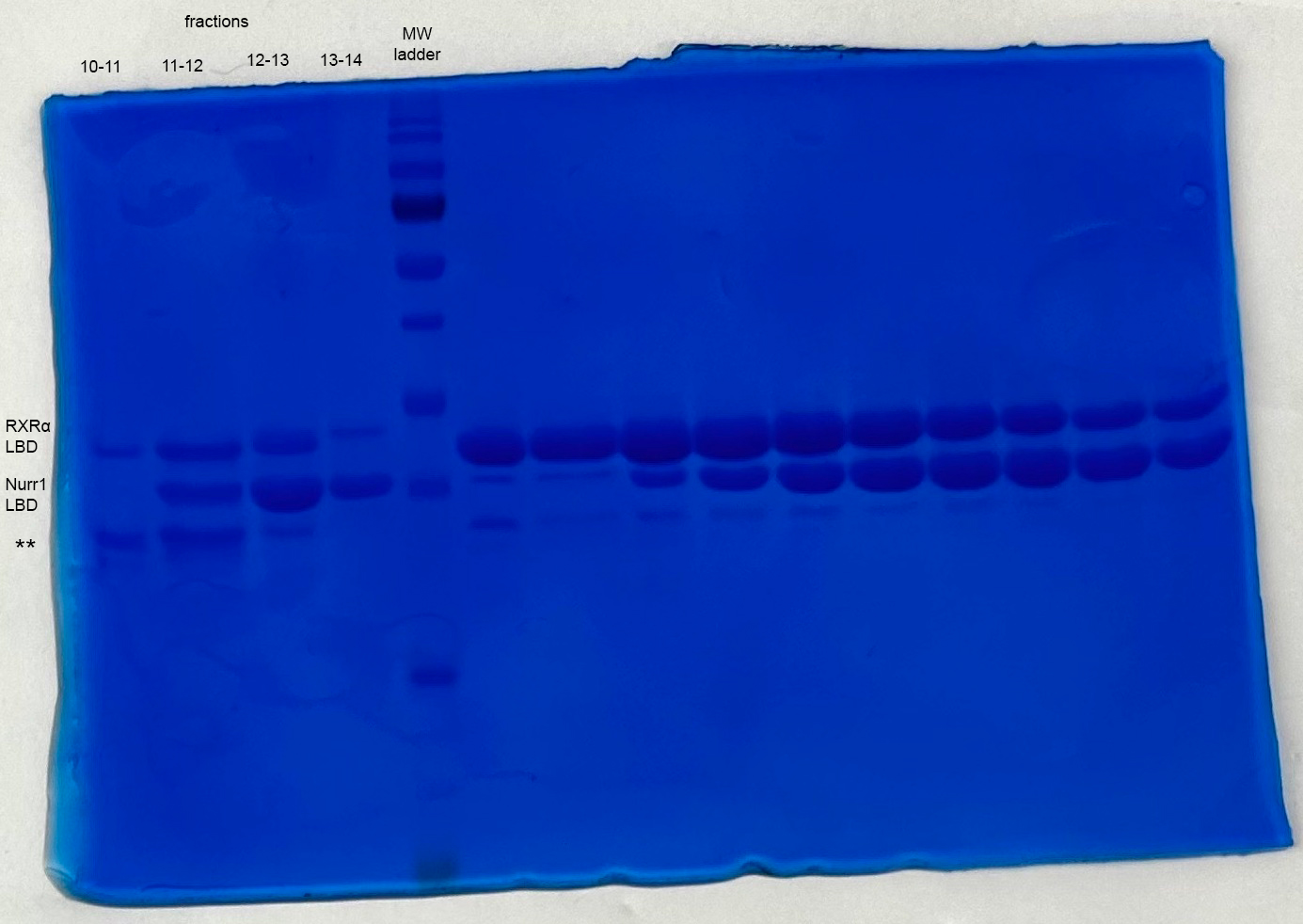

Supplement: Figure 6—figure supplement 3—source data 1. [file elife-85039-fig6-figsupp3-data1.zip › Figure 6-figure supplement 3-source data 1/full raw unedited gel annotated.jpg]

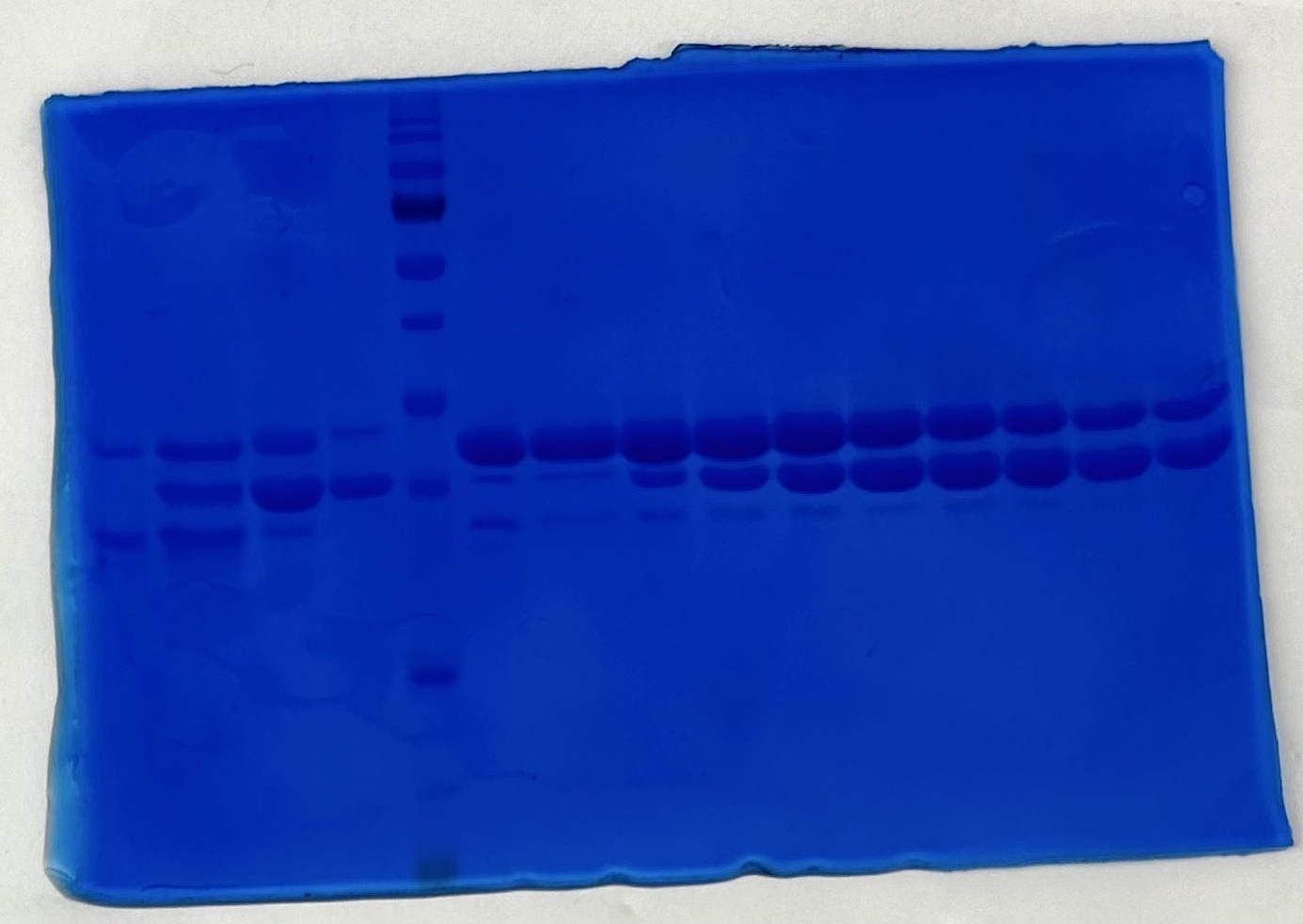

Supplement: Figure 6—figure supplement 3—source data 1. [file elife-85039-fig6-figsupp3-data1.zip › Figure 6-figure supplement 3-source data 1/full raw unedited gel.jpg]
