# Supplementary material for: Molecular basis of ligand-dependent Nurr1-RXRα activation
Source: eLife. 2023 Apr 27;12:e85039. doi: 10.7554/eLife.85039 (PMC10259986; doi:10.7554/eLife.85039)
Supplement: Supplementary file 1. [file elife-85039-supp1.docx]

**Supplementary File 1. *gBlock sequence used to clone the RXRα LBD only construct***

CCAGCACAGTGGCGGCCGCATGAAGCGGGAAGCCGTGCAGGAGGAGCGGCAGCGTGGCAAGGACCGGAACGAGAATGAGGTGGAGTCGACCAGCAGCGCCAACGAGGACATGCCGGTGGAGAGGATCCTGGAGGCTGAGCTGGCCGTGGAGCCCAAGACCGAGACCTACGTGGAGGCAAACATGGGGCTGAACCCCAGCTCGCCGAACGACCCTGTCACCAACATTTGCCAAGCAGCCGACAAACAGCTTTTCACCCTGGTGGAGTGGGCCAAGCGGATCCCACACTTCTCAGAGCTGCCCCTGGACGACCAGGTCATCCTGCTGCGGGCAGGCTGGAATGAGCTGCTCATCGCCTCCTTCTCCCACCGCTCCATCGCCGTGAAGGACGGGATCCTCCTGGCCACCGGGCTGCACGTCCACCGGAACAGCGCCCACAGCGCAGGGGTGGGCGCCATCTTTGACAGGGTGCTGACGGAGCTTGTGTCCAAGATGCGGGACATGCAGATGGACAAGACGGAGCTGGGCTGCCTGCGCGCCATCGTCCTCTTTAACCCTGACTCCAAGGGGCTCTCGAACCCGGCCGAGGTGGAGGCGCTGAGGGAGAAGGTCTATGCGTCCTTGGAGGCCTACTGCAAGCACAAGTACCCAGAGCAGCCGGGAAGGTTCGCTAAGCTCTTGCTCCGCCTGCCGGCTCTGCGCTCCATCGGGCTCAAATGCCTGGAACATCTCTTCTTCTTCAAGCTCATCGGGGACACACCCATTGACACCTTCCTTATGGAGATGCTGGAGGCGCCGCACCAAATGACTTGATCGAGTCTAGAGGGCCCG
